# A transparent window into the rhizosphere: a simplified workflow for spatially resolved soil metabolomics

**DOI:** 10.1093/aobpla/plag055

**Published:** 2026-10-30

**Authors:** Harihar Jaishree Subrahmaniam, Paul Moritz, Phillip J Becker, Maria Riedner, Simon Thomsen, Ina C Meier, Kai Jensen

**Affiliations:** Applied Plant Ecology , Institut für Pflanzenwissenschaften und Mikrobiologie (IPM), University of Hamburg, Ohnhorststraße 18, Hamburg 22609, Germany; Functional Forest Ecology, Institut für Pflanzenwissenschaften und Mikrobiologie (IPM), University of Hamburg, Ohnhorststraße 18, Hamburg 22609, Germany; Applied Plant Ecology , Institut für Pflanzenwissenschaften und Mikrobiologie (IPM), University of Hamburg, Ohnhorststraße 18, Hamburg 22609, Germany; Functional Forest Ecology, Institut für Pflanzenwissenschaften und Mikrobiologie (IPM), University of Hamburg, Ohnhorststraße 18, Hamburg 22609, Germany; Technology Platform Mass Spectrometry, University of Hamburg, Martin-Luther-King-Platz 6, Hamburg 20146, Germany; Applied Plant Ecology , Institut für Pflanzenwissenschaften und Mikrobiologie (IPM), University of Hamburg, Ohnhorststraße 18, Hamburg 22609, Germany; Functional Forest Ecology, Institut für Pflanzenwissenschaften und Mikrobiologie (IPM), University of Hamburg, Ohnhorststraße 18, Hamburg 22609, Germany; Applied Plant Ecology , Institut für Pflanzenwissenschaften und Mikrobiologie (IPM), University of Hamburg, Ohnhorststraße 18, Hamburg 22609, Germany

**Keywords:** 3D-printed rhizobox, LC-MS, soil metabolomics, root–root interactions, abiotic and biotic stress

## Abstract

Root exudates shape nutrient cycling, microbial recruitment, and plant–plant interactions, yet most approaches for analysing exudate chemistry rely on hydroponic systems that poorly represent soil. We present an open-source workflow built around a 3D-printed rhizobox that enables non-destructive root imaging and targeted rhizosphere soil sampling for LC-MS metabolomics. The workflow combines a frame that prints as a single piece on a consumer printer, sampling of defined soil zones registered to root images, and a blank-informed, feature-specific background filter. The filter is essential because ∼90% of detected features also occur in soil, material or solvent controls. In a validation experiment with *Arabidopsis thaliana* and *Phragmites australis*, both species grew along the window, and species identity explained 38% of compositional variation among rhizoboxes, whereas sampling date and depth had no detectable effect. In a factorial experiment with *A. thaliana*, rhizoboxes holding intraspecific pairs showed higher metabolite richness and diversity and a distinct composition compared with solo plants, whereas short heat stress had no detectable effect. Inner and outer sampling zones of paired boxes did not differ at the replication available, and we give the replication needed to resolve such effects. The protocol can be implemented within 4 to 6 weeks using standard laboratory equipment and openly available design files. Combining ecological realism with analytical control, this workflow offers a practical route to quantifying rhizosphere metabolite dynamics across species, treatments, and defined sampling zones, facilitating experimental study of below-ground processes in plant ecology.

## Teaser

Plants release root chemicals that shape soil life and interactions with neighbours, but these are hard to measure in real soil. We present a low-cost, 3D-printed box with a clear window that lets researchers watch roots grow and sample the soil right beside them. With it, two plant species could be told apart by their soil chemistry, and Arabidopsis plants grown with a neighbour left a richer chemical mixture than plants grown alone.

## Introduction

Plants actively modify their below-ground environment through the release of a diverse array of low-molecular-weight compounds, including sugars, amino acids, organic acids, and secondary metabolites. These root-derived compounds contribute to nutrient mobilization, microbial recruitment, and plant–plant interactions, linking plant physiology to soil ecological processes (Bais et al. 2006, Mommer et al. 2016, Canarini et al. 2019, Anten and Chen 2021, Ma et al. 2022). Despite their recognized importance, experimental investigation of root exudate chemistry remains methodologically constrained, particularly under realistic soil conditions (Oburger and Jones 2018, Escolà Casas and Matamoros 2021). Root exudation occurs within an opaque, microbially active, and spatially heterogeneous matrix, where metabolites are rapidly transformed and diluted. Consequently, detection and interpretation of exudate-derived signals depend strongly on sampling position relative to roots and on the ability to distinguish plant-derived compounds from soil background (Ritter et al. 2025). Methods that enable consistent, spatially explicit rhizosphere sampling while preserving soil structure are therefore essential for linking chemical profiles to plant traits and experimental treatments over time.

Experimental studies of plant–plant interactions increasingly recognize that below-ground chemical cues contribute to neighbour detection, kin recognition, and stress context-dependent responses (Dudley and File 2007, Biedrzycki et al. 2010, Semchenko et al. 2014, Subrahmaniam et al. 2018). However, most empirical work in this area still relies on hydroponic, agar-based, or split-root systems. Soil-based methods, including cuvette-based *in situ* exudate collection, whole-root dipping approaches and sorption-trap sampling approaches, reduce this gap but still involve disturbance or short-term collection windows, constraining spatially explicit metabolomic analyses. As a result, it remains difficult to experimentally address where in the soil matrix chemical interactions occur and how metabolite profiles vary across neighbouring root zones under controlled yet realistic conditions.

Abiotic stressors such as heat and drought induce pronounced metabolic adjustments in plants, which often involve shifts towards osmoprotectants, antioxidants, and phenolic compounds (Bita and Gerats 2013, Gargallo-Garriga et al. 2018). These changes can propagate into the rhizosphere, influencing microbial activity and chemical composition of soil adjacent to roots (Rolfe et al. 2019). Importantly, stress responses may depend on social context, with neighbour presence altering resource allocation or modulating chemical outputs through facilitative or competitive interactions (Vives-Peris de Ollas et al. 2020, Pang et al. 2021, Sharma et al. 2023). Despite growing interest in biotic × abiotic interactions below ground, very few experimental systems allow simultaneous manipulation of neighbour identity and environmental stress while remaining compatible with untargeted metabolomic analysis in soil (Ritter et al. 2025).

Applying LC-MS-based metabolomics to soil systems remains technically demanding. Hydroponic and agar-based approaches provide clean matrices and high analytical sensitivity, but they lack realistic diffusion gradients and microbial turnover (Oburger and Jones 2018). In contrast, although soil-based experiments offer ecological relevance, they introduce a background signal from soil organic matter, growth substrates and experimental materials that dominates the measured profile rather than merely adding to it, so the plant-derived component is a minor constituent of an untargeted soil extract. A further challenge lies in spatial control. While many existing rhizobox designs prioritize root imaging, they were not developed for large-scale, replicated, and treatment-rich metabolomics experiments. They often lack fixed sampling geometries (i.e. predefined soil volumes and distances from roots), consistent sampling interfaces, and systematic blank controls, all of which are essential for scalable chemical analyses. Although recent hydroponic workflows have standardized exudate collection for metabolomics (Subrahmaniam et al. 2023, Döll et al. 2024), systems that integrate root visibility, spatially defined soil sampling and blank-informed metabolomic workflows in a single openly available and documented package remain uncommon, since in-house variants are seldom released.

Whereas hydroponic systems quantify compounds released into solution, soil-based sampling captures the pool of exudate-derived metabolites that persist, are transformed or accumulate in the rhizosphere, which is the chemical environment that neighbouring roots and microbes actually encounter. Rhizoboxes, growth containers with transparent windows for root observation, have a long history in root research, and many laboratories build their own variants. Recent systems combine root imaging with chemical sampling. RhizoMAP images metabolites imprinted onto membranes by MALDI mass spectrometry (Veličković et al. 2024), and sorption traps placed against roots capture exudates for GC-HRMS (Bohm et al. 2025). These approaches offer high spatial resolution or root-surface specificity. However, they require specialized instrumentation or sample a different chemical pool, and they are not designed for replicated factorial experiments with per-sample LC-MS profiles. Our contribution is therefore not the rhizobox itself but the integration of three elements: a frame that prints as a single piece on a consumer printer; soil sampling from defined zones registered to a root image; and a blank-informed, feature-specific filter that separates plant-derived signal from soil, material, and solvent background. We release the design openly because the barrier to adoption is usually not the concept but the availability of a file that prints, seals and can be sampled reproducibly.

To address these limitations, we developed an open-source, 3D-printed rhizobox optimized for rhizosphere metabolomics in soil. The system incorporates a transparent, removable front window that enables repeated, non-destructive imaging of root systems and guides targeted rhizosphere soil sampling. Defined geometry and modular components support reproducible sampling positions across replicates and experimental treatments.

In this study, we present and validate an experimental workflow for quantifying rhizosphere metabolite dynamics in soil using the 3D-printed rhizobox system. Specifically, our aims are:

1. Demonstrate that the rhizobox supports consistent plant establishment and root development under soil conditions, and the workflow yields a reproducible plant-associated feature set despite a dominant soil and material background.
2. Assess cross-species applicability by comparing the rhizosphere metabolite profiles of two contrasting species, the small rosette model *Arabidopsis thaliana* (Col-0) and the large non-model wetland grass *Phragmites australis*; and
3. Evaluate sensitivity to biologically relevant treatments by conducting a factorial experiment manipulating social context (solo vs. intraspecific pairs) and short-term heat stress and using the paired inner and outer sampling zones of the pair treatment to test for spatial structure within a single rhizobox.

By detailing the design, sampling strategy, and analytical workflow, this study provides a scalable and accessible framework for integrating metabolomics into experimental plant ecology.

## Materials and methods

### Overview

Two experiments were conducted. Experiment 1 (April–July 2025) validated the rhizobox with *A. thaliana* (Col-0) and *P. australis*, sampled on three dates and at two depths. Experiment 2 (October–November 2025) applied the workflow to a factorial manipulation of social context (solo vs. intraspecific pair) and air temperature (ambient vs. short-term heat stress) in *A. thaliana* (Col-0) including zone-resolved spatial sampling.

### Rhizobox design and assembly

Rhizoboxes were produced from a high-flow variant of polyethylene terephthalate glycol (PETG-HF) filament (Bambu Lab, Shenzhen, China), using a Bambu Lab X1E Fused Deposition Modeling 3D printer (Bambu Lab, Shenzhen, China). All G-code was generated using Bambu Studio (v1.10.2.64, Bambu Lab). The rhizoboxes consisted of an opaque frame measuring 97 × 94 × 10 mm internally, with a perforated base to allow drainage. The 1 cm soil thickness keeps the whole soil volume within a few millimetres of roots and within the depth of field of the window. The transparent front window was made from a 2 mm-thick sheet of PLEXIGLAS^®^ XT (extruded acrylic glass, PMMA, Röhm GmbH, Germany) and was attached using metal clamps. This enabled root imaging and access for exudate sampling. To exclude light, the window was fully covered with a removable 0.3 mm thick, opaque PVC sheet, with the edges sealed with matt black tape. Boxes were mounted at 45° from the vertical on a custom frame to direct root growth. The design files are provided as Supplementary Data S1 (print file) and S2 (dimensioned schematic). Material cost is EUR 5.79 per rhizobox at single-unit prices, comprising PETG-HF filament (EUR 1.37 for 55 g), the PMMA window (EUR 3.92), and the opaque PVC cover (EUR 0.50). Because sheet material attracts volume discounts while filament does not, unit cost falls to EUR 3.20 when 10 boxes are built together and EUR 2.73 at one hundred, with the window accounting for 68% of the cost of a single unit but only 32% at one hundred. Costs are given in EUR for Germany in 2025 and exclude the reusable metal clamps, the printer as capital equipment, electricity, and substrate.

All components were rinsed with 96% ethanol and air-dried prior to use to remove surface residues; the system was not maintained sterile, allowing normal microbial recolonization from soil. The inner surface of the Plexiglas front panel was coated with a thin layer of polytetrafluoroethylene (PTFE) spray to reduce soil adhesion and facilitate consistent rhizosphere sampling; PTFE is chemically inert and was confined to the window surface, minimizing direct contact with roots or soil.

### Soil preparation and greenhouse conditions

A standard peat-based soil (Einheitserde Type ED73; Einheitserde Werkverband, Germany) was sieved to remove coarse particles and homogenized. Rhizoboxes were loosely packed to a consistent fill height and moistened evenly with sterile distilled water prior to sealing the window. Throughout experiments, moisture was maintained by surface watering using a spray bottle to avoid waterlogging. No fertilizer was applied, as the aim was to maintain uniform, moderate nutrient conditions and avoid treatment-independent shifts in rhizosphere chemistry. The experiments were conducted in a climate-controlled greenhouse at the host institution under the following conditions: air temperature 22 ± 1°C, 16 h light/8 h dark photoperiod, and a photosynthetically active radiation of 120–150 µmol m^−2^ s^−1^.

### Plant cultivation

Seeds of *A. thaliana* were stratified for 72 h at 4°C in darkness, germinated at 22°C, and transplanted into rhizoboxes at the cotyledon stage using a moistened fine brush. Plants of *P. australis* were propagated in trays under greenhouse conditions and transferred into rhizoboxes at comparable early developmental stages to *A. thaliana*.

### Experimental designs and rhizosphere sampling

#### Experiment 1

To validate the rhizobox system for soil-based metabolomics, six rhizoboxes were established for each of two species (*A. thaliana* Col-0 and *P. australis*), together with rhizoboxes containing soil but no plant, with and without PTFE coat on the window (Supplementary Table S1). Plants were grown for 3 weeks and sampled on three dates (13 June, 18 June, and 23 June 2025). At each sampling date, root systems were imaged by removing the transparent front panel. Immediately after imaging, rhizosphere soil was collected using sterile spatulas from within ∼5 mm of visible roots to ensure close root proximity. Approximately 0.3 g of soil per sample was transferred to 2 ml tubes, kept on ice during processing, and stored at −80°C until extraction. Sampling targeted two predefined vertical positions within each rhizobox: an upper soil zone (∼0–5 cm below the surface) and a lower soil zone (∼5–10 cm). To minimize disturbance artefacts from repeated sampling, soil was collected from different sides of the visible root system across time points.

#### Experiment 2

To test the effects of social context and short-term heat stress on rhizosphere metabolomes, *A. thaliana* (Col-0) was grown under three configurations: (i) solo (one plant per rhizobox), (ii) intraspecific pair (two plants per rhizobox, spaced ∼2 cm apart), and (iii) rhizoboxes with soil but no plant. Seeds were sown in early October 2025 and transplanted into rhizoboxes at the cotyledon stage. Plants were grown under greenhouse conditions for ∼4 weeks until the late vegetative rosette stage. Each configuration was exposed either to ambient conditions (22°C) or to acute heat stress (45°C for 2 h in a pre-heated chamber), followed by a 2 h recovery period at 22°C. Five biological replicates were established per treatment × temperature combination (20 planted rhizoboxes total, 6 unplanted controls). All box positions were fully randomized within the greenhouse. Immediately after treatment, root systems were imaged, and rhizosphere soil was collected using the same procedure as in Experiment 1. In pair treatments, sampling was spatially explicit: soil was collected separately from the zone between the two root systems, hereafter the inner zone, and from the zones lateral to the pair, hereafter the outer zone, giving paired inner and outer samples from the same rhizobox. Zone-resolved sampling was possible only in the paired boxes, since the inner zone is defined by the presence of two plants; three boxes therefore contribute zone-resolved samples under each temperature condition, with two inner and two outer samples per box (four zone samples per box).

Owing to plant loss, four *A. thaliana* and six *P. australis* rhizoboxes were analysed in Experiment 1, and three rhizoboxes per social context × temperature combination in Experiment 2 (Supplementary Table S1).

### Phenotypic measurements and root imaging

Plants were photographed at each sampling date in Experiment 1 and once prior to treatment and sampling in Experiment 2. Aboveground traits (rosette diameter and leaf number) were measured using digital callipers. Roots were imaged by removing the transparent front panel and using a digital camera under greenhouse lighting. Root length and surface area were quantified in ImageJ/Fiji using the SmartRoot plugin (Lobet et al. 2011), with pixel-to-millimetre calibration based on a ruler included in each image. Open-source alternatives for batch quantification of root images, such as RhizoVision Explorer (Seethepalli et al. 2021), are equally compatible with the image series the system produces and would be preferable where large numbers of images require standardized measurement.

### Material and background controls

To account for background from the soil matrix, construction materials and solvents, control samples were extracted, measured and processed alongside the planted samples and used to define the background filter (‘Data processing, blank-informed filtering, and annotation’ section). In Experiment 1, controls were rhizoboxes with soil but no plant, with and without PTFE coat on the window, and solvent blanks. In Experiment 2, glass–soil controls tested for material- and heat-induced artefacts, alongside rhizoboxes with soil but no plant and solvent blanks. Material-associated features were detected in these controls, as expected for any polymer surface in contact with solvent, but none was retained after filtering.

### Metabolite extraction

Metabolites were extracted from frozen soil following a modified protocol based on Pétriacq et al. (2017) (Supplementary Data S4). Briefly, 0.2 g soil (*A. thaliana*) and 0.3 g soil (*P. australis*, Experiment 1), and 0.2 g soil in Experiment 2, were mixed with 1 ml pre-cooled extraction solvent (methanol:water:formic acid, 80:19.9:0.1, v/v/v), vortexed for 30 s, and shaken horizontally for 45 min at 800 rpm and 4°C. Samples were centrifuged at 14 000 × *g* for 15 min (4°C), and supernatants were filtered through 0.22 µm PTFE filters into LC-MS vials and stored at −20°C until analysis. All control samples were extracted in parallel using identical procedures. Because soil particles contain adsorption and ion-exchange sites, a fraction of root-derived metabolites may be retained or transformed within the soil matrix. The extraction therefore captures the pool of metabolites that are recoverable under the applied solvent conditions, representing the detectable rhizosphere metabolite fraction rather than the total amount of compounds released by roots.

### LC-MS acquisition

LC-MS data were acquired on a Bruker maXis QTOF coupled to a Bruker Elute UHPLC. Chromatography was performed on a Waters BEH C18 column (2.1 × 100 mm, 1.8 µm) at 50°C. Mobile phase A was 0.1% formic acid in water and mobile phase B was 0.1% formic acid in acetonitrile. Mobile phase B was held at 2% for the first 3 min, afterwards, the gradient increased from 2% to 90% B over 10 min, was held for 1.5 min, and re-equilibrated at 2% B for 4 min (flow rate 0.5 ml min^−1^). The injection volume was 5 µl per sample. The QTOF operated in positive electrospray (ESI) mode (capillary voltage 4.5 kV; source temperature 200°C; drying gas 9 l min^−1^; scan range *m/z* 50–1500), with auto-MS/MS acquisition of the top five ions per cycle. Pooled QC samples were injected every ten runs to monitor retention time and signal stability, and solvent blanks confirmed the absence of carry-over. QC ordination showed one lower-volume injection and a gradual signal decline across the run; because injection order was randomized with respect to treatment and the filter evaluates presence relative to controls, this drift does not bias group comparisons.

### Data processing, blank-informed filtering, and annotation

Raw vendor files (.d) were converted to the open mzML format using ProteoWizard (Chambers et al. 2012). Converted files were processed in MZmine 3 (Schmid et al. 2023) using identical parameter settings across all samples and experiments to ensure comparability. Processing steps included mass detection, chromatogram building, ADAP wavelet-based peak deconvolution, isotopic grouping, retention-time alignment, and gap filling (Supplementary Data S5). Feature tables and associated metadata were exported to R for downstream analysis. Signal intensities were treated as numeric values, with zero intensities retained to preserve true absence information. Data were log-transformed to stabilize variance and reduce the influence of extreme values while maintaining sensitivity to low-abundance features. Where required for interpretation or summary, linear intensities were reconstructed from transformed values.

Background signal was removed feature by feature. For each feature, a limit of detection (LOD) was defined from the control samples as the median control intensity plus 2.5 times the median absolute deviation. In Experiment 1, a feature was retained if it was not detected in any control sample and exceeded its LOD in at least three rhizoboxes of one species. In Experiment 2, a feature was retained if its maximum control intensity did not exceed the LOD, its maximum sample intensity was at least three times the maximum control intensity, and it exceeded the LOD in at least three rhizoboxes of one temperature condition. Prevalence was counted per rhizobox rather than per sample, because each box was sampled repeatedly. In Experiment 2, solvent blanks and glass–soil controls informed the LODs of both temperature conditions, and soil-only rhizoboxes were matched to their own condition. A whole-profile criterion could not be used. In Experiment 1, 1245 of 1358 detected features (91.7%) also occurred in at least one control and carried on average 99.8% of the total ion signal of a planted sample, so soil-only controls overlapped planted samples in unfiltered ordinations (Supplementary Figure S1). Because thresholds were derived from each experiment’s own controls, retained feature sets and the metrics computed from them are not comparable in absolute terms between experiments.

Putative compound annotation was performed using MS/MS-based analysis in SIRIUS 4 (Dührkop et al. 2019). Molecular formulae were predicted from fragmentation trees, and candidate structures were inferred using CSI:FingerID (Dührkop et al. 2015). Chemical class assignments were obtained using CANOPUS (Dührkop et al. 2021)/NPClassifier (Kim et al. 2021), enabling pathway-level interpretation even when exact structural identification was not possible. SIRIUS outputs were linked to MZmine features using explicit feature identifiers. In cases where multiple annotations mapped to a single feature, the annotation with the highest confidence and information content was retained. Curated annotations were used exclusively for class-level and pathway-based analyses.

### Statistical analysis

Analyses were run in R 4.5.2 (R Core Team 2025) using vegan 2.7.5 (Oksanen et al. 2025) and permute; scripts are provided as Supplementary Data S3. For each sample we calculated total signal (summed intensity of retained features), feature richness and Shannon diversity of relative intensities. The rhizobox was the unit of replication. Factors varying between boxes (species, social context, temperature) were tested on one value per box. These were box means for summary metrics and log2 feature intensities, and, for composition in Experiment 1, the presence of each feature in any sample of the box. Factors varying within boxes (sampling date, depth, inner vs. outer zone) were tested on samples, with permutations restricted within box. Composition was analysed by PERMANOVA (adonis2; sequential terms; 9999 permutations) on Jaccard distances of presence–absence profiles, and multivariate dispersion with betadisper. In Experiment 2, paired boxes contributed four zone samples and solo boxes one. Between-box compositional and feature-wise tests were therefore effort-matched: one zone sample was drawn at random per paired box, the test was run on the 12 boxes, and medians over 999 draws are reported. Summary metrics were analysed with linear models on box means (Experiment 2: two-way ANOVA of social context × temperature). Feature-wise differences were tested with linear models (Experiment 1) or *t*-tests (Experiment 2) on boxes, with Benjamini–Hochberg false discovery rate (FDR) control. Pathway composition was expressed as the share of a sample’s retained signal assigned to each NPClassifier pathway, averaged per box. Inner and outer zones were compared with paired *t*-tests across paired boxes, for which we report the minimum difference detectable at 80% power. Principal component analysis (PCA) of log2 intensities and metric multidimensional scaling (MDS) of Bray–Curtis distances were used for visualization.

## Results

### System performance and LC-MS data quality across experiments

Surviving plants of both species developed root systems that grew along the transparent window, allowing repeated imaging and targeted soil sampling (Fig. 1b). No rhizobox showed waterlogging, wilting or avoidance of the window, and soil moisture was kept similar across treatments, so the heat treatment was not confounded with drought. In unfiltered ordinations, solvent blanks separated from all other samples, whereas soil-containing controls overlapped the planted samples (Supplementary Figure S1). The blank-informed filter retained 20 of 1358 features in Experiment 1 (15 detected in *A. thaliana*, 12 in *P. australis*; Supplementary Table S2) and 28 of 583 features in Experiment 2 (27 under ambient conditions and 15 under heat, 14 of them shared; Supplementary Table S3).

**Figure 1:**
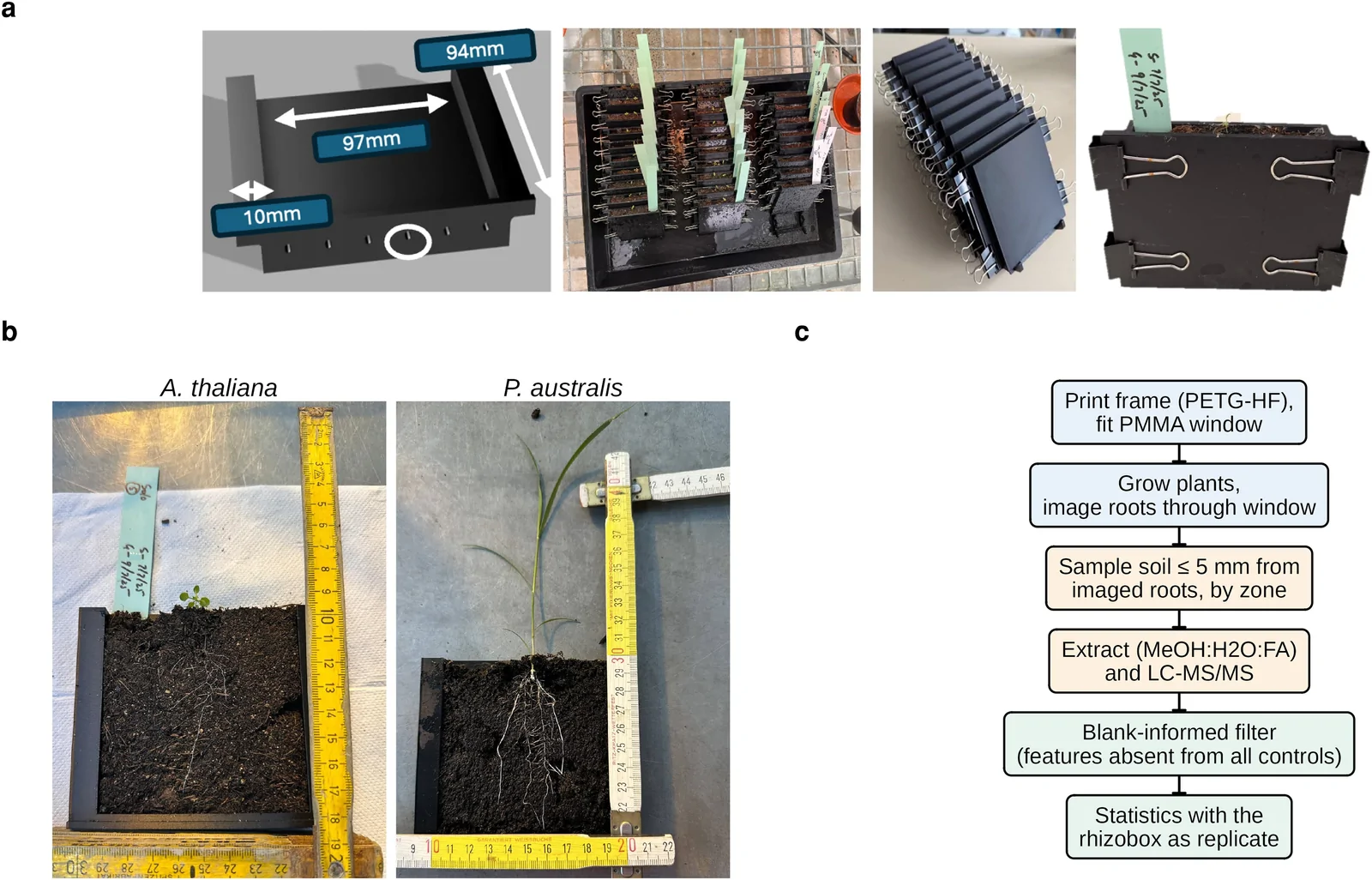
The rhizobox system and workflow. (a) Rhizobox design, from left: dimensioned render of the printed frame (internal 97 × 94 × 10 mm); rhizoboxes in a tray during Experiment 1; stacked boxes with windows clamped; a single box with the light-excluding cover. (b) Root systems of *Arabidopsis thaliana* and *Phragmites australis* iwith the window removed; the rulers give scale (cm). (c) Workflow from printing to statistics.

### Species-specific rhizosphere metabolite profiles in *A. thaliana* and *P. australis* (Experiment 1)

Rhizosphere metabolite composition differed clearly between species. *A. thaliana* and *P. australis* separated along the first principal component (Fig. 2a), and species explained 38% of compositional variation among rhizoboxes (PERMANOVA, *R*^2^ = 0.38, *F*_1,8_ = 4.82, *P* = .005; Supplementary Table S4). The species also differed in multivariate dispersion (*F*_1,8_ = 5.90, *P* = .036), with *P. australis* boxes more heterogeneous, so part of the separation reflects spread as well as mean composition. Within rhizoboxes, composition was affected neither by sampling date (*R*^2^ = 0.05, *F*_2,30_ = 1.21, *P* = .24) nor by its interaction with species (*F*_2,30_ = 1.10, *P* = .33), nor by depth (*R*^2^ = 0.01, *F*_1,30_ = 0.50, *P* = .86). *A. thaliana* rhizoboxes had higher total signal than *P. australis* rhizoboxes (8843 ± 1057 vs. 1918 ± 1109; *F*_1,8_ = 18.3, *P* = .003). They also had higher feature richness (7.8 ± 0.2 vs. 1.9 ± 0.5; *F*_1,8_ = 80.7, *P* < .001) and Shannon diversity (1.70 ± 0.05 vs. 0.46 ± 0.14; *F*_1,8_ = 44.8, *P* < .001; Fig. 2b–d; Supplementary Table S5). Fifteen of the 20 features differed between species after FDR correction (Supplementary Table S2). Retained features were assigned to alkaloids, amino acids and peptides, fatty acids, polyketides, shikimates and phenylpropanoids, terpenoids, and unassigned compounds (Fig. 3a). Fatty acids accounted for a 20 percentage-point larger share of signal in *A. thaliana* than in *P. australis* (*P* = .009), although this difference was not significant after FDR correction (FDR = 0.066). The other pathway shares did not differ between species (Fig. 3b) and were broadly stable across sampling dates (Fig. 3c).

**Figure 2:**
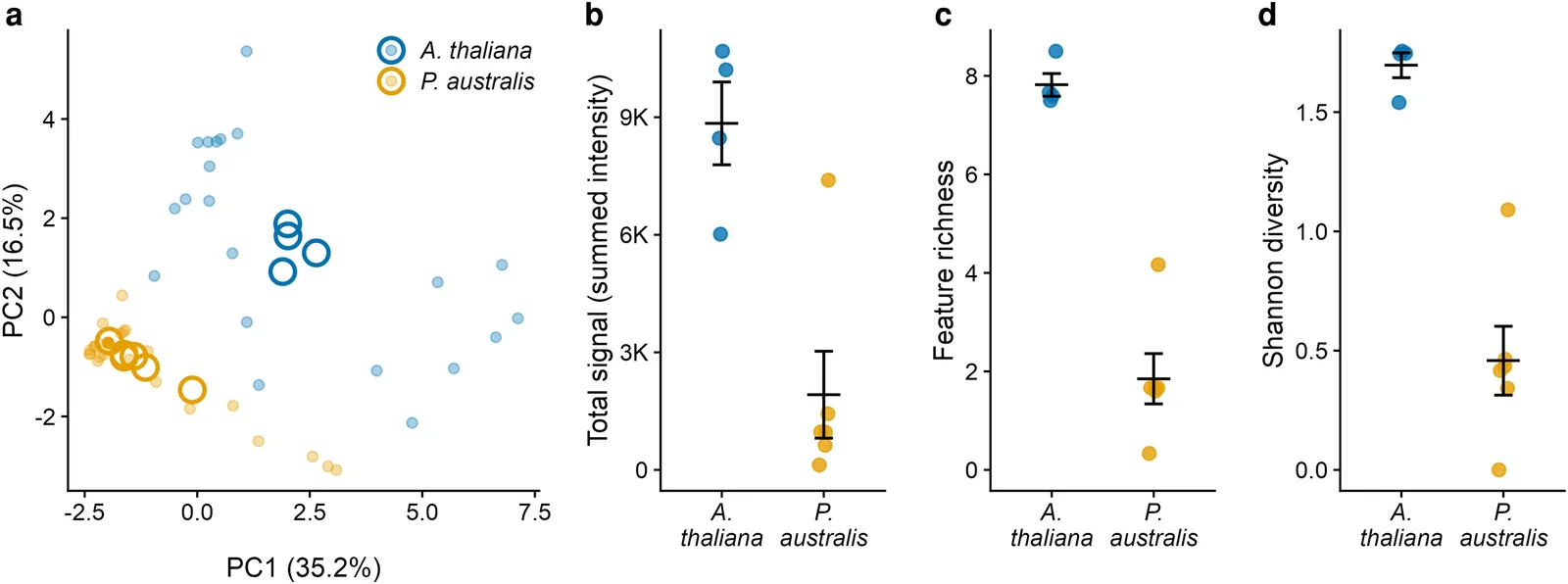
Rhizosphere metabolome differences between *A. thaliana* and *P. australis* (Experiment 1; 20 retained features). (a) Principal component analysis of log2-transformed feature intensities. Small points are samples; large open circles are rhizobox means. (b) Total signal, (c) feature richness and (d) Shannon diversity. Points are rhizobox means of the per-sample values (4 *A. thaliana*, 6 *P. australis* boxes); bars show mean ± SE across boxes.

**Figure 3:**
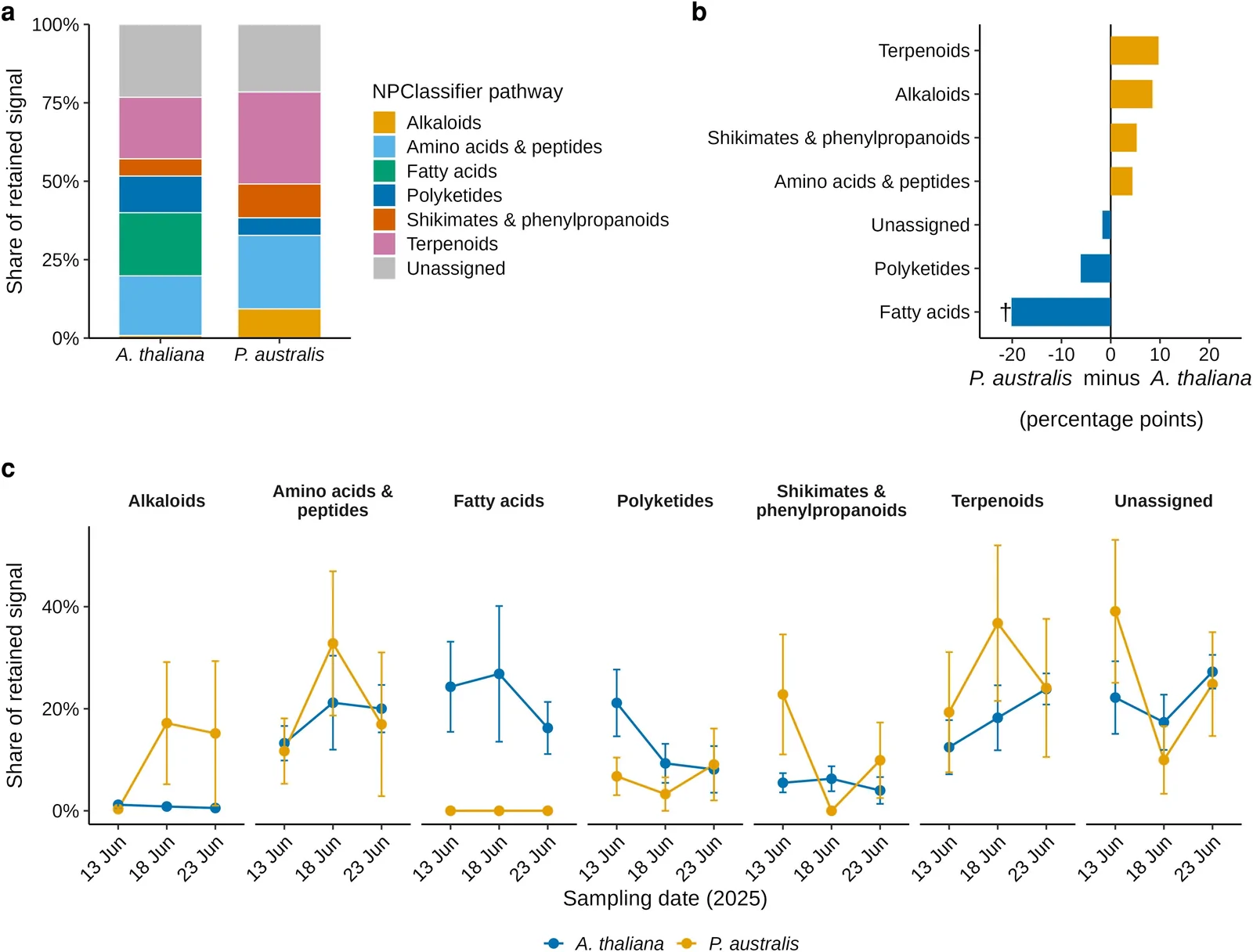
Chemical class composition in Experiment 1. (a) Mean share of retained signal per NPClassifier pathway. Shares were computed per sample, averaged per rhizobox, then across boxes. (b) Difference in pathway share between species (*P. australis* minus *A. thaliana*, percentage points). ^†^*P* < .05 before, but not after, FDR correction (linear model on rhizobox means). (c) Pathway shares on the three sampling dates (mean ± SE across samples).

### Effects of social context and heat stress on Arabidopsis rhizosphere metabolomes (Experiment 2)

Social context structured the rhizosphere metabolome of *A. thaliana* more strongly than heat did (Fig. 4a). Across rhizoboxes, social context explained 27% of compositional variation (*R*^2^ = 0.27, *F*_1,8_ = 4.01, *P* = .011; significant in all 999 effort-matched draws). Temperature (*R*^2^ = 0.12, *F*_1,8_ = 1.78, *P* = .13) and the interaction (*F*_1,8_ = 0.66, *P* = .66) had no detectable effect (Supplementary Table S4). Paired rhizoboxes had higher feature richness than solo rhizoboxes (ambient: 16.2 ± 1.7 vs. 4.7 ± 1.2; heat: 11.6 ± 1.6 vs. 5.0 ± 0.6; *F*_1,8_ = 46.8, *P* < .001). They also had higher Shannon diversity (ambient: 2.12 ± 0.21 vs. 1.02 ± 0.22; heat: 1.68 ± 0.15 vs. 1.16 ± 0.17; *F*_1,8_ = 17.9, *P* = .003). Temperature and the interaction did not affect either metric (all *P* ≥ .097; Fig. 4b–d; Supplementary Table S5). Total signal did not differ between social contexts (*F*_1,8_ = 3.84, *P* = .086) or temperatures (*F*_1,8_ = 0.05, *P* = .83). Three features were more abundant in paired than in solo rhizoboxes under ambient conditions, and one feature increased under heat in solo rhizoboxes (FDR < 0.05; Supplementary Table S3). Retained features were fatty acids, amino acids and peptides, alkaloids, and unassigned compounds. Fatty acids dominated all treatments, and heat-shifted pathway shares more in solo than in paired rhizoboxes (Fig. 5).

**Figure 4:**
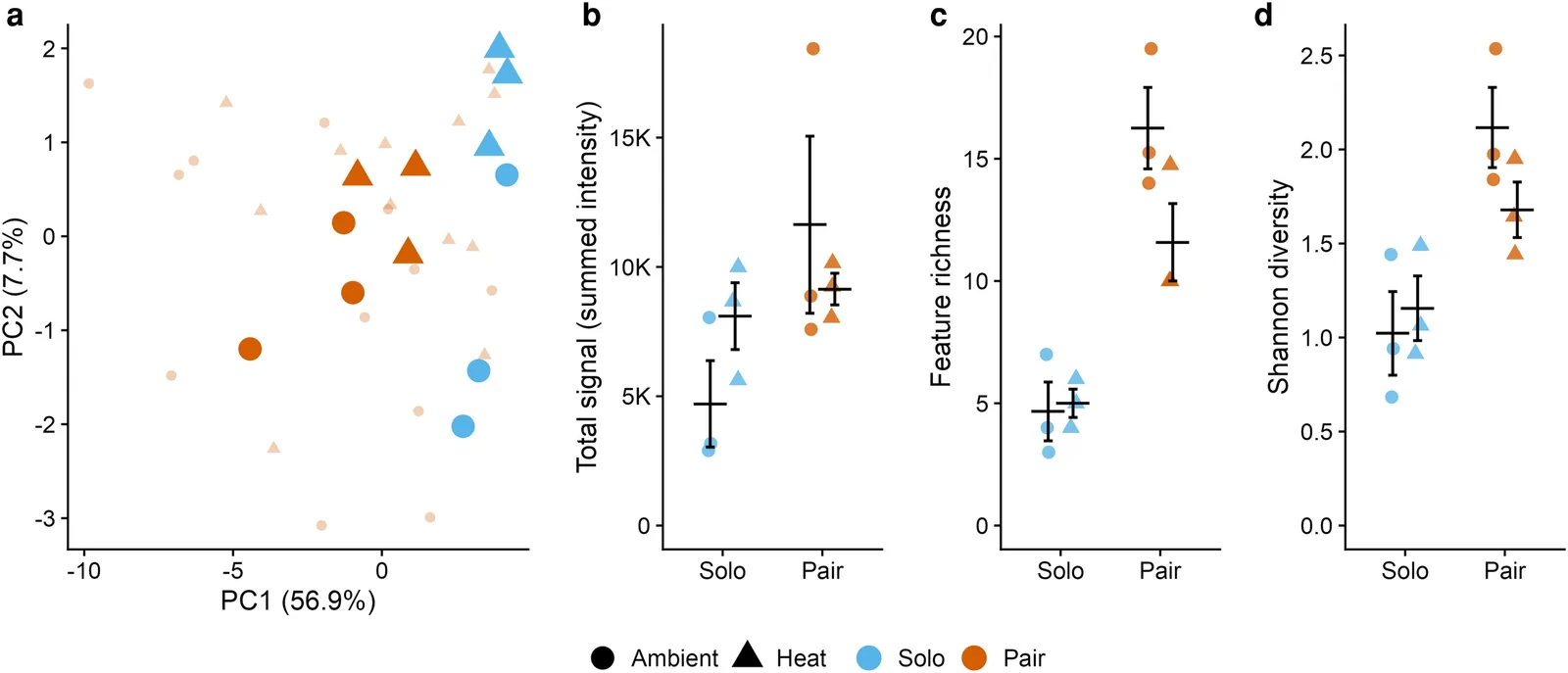
Effects of social context and short-term heat on *A. thaliana* rhizosphere metabolomes (Experiment 2; 28 retained features). (a) PCA of log2 feature intensities. Small points are samples; large points are rhizobox means; colour gives social context and shape gives temperature. (b) Total signal, (c) feature richness and (d) Shannon diversity. Points are rhizobox means (paired boxes: mean of four zone samples); bars show mean ± SE across the three boxes per treatment × temperature combination.

**Figure 5:**
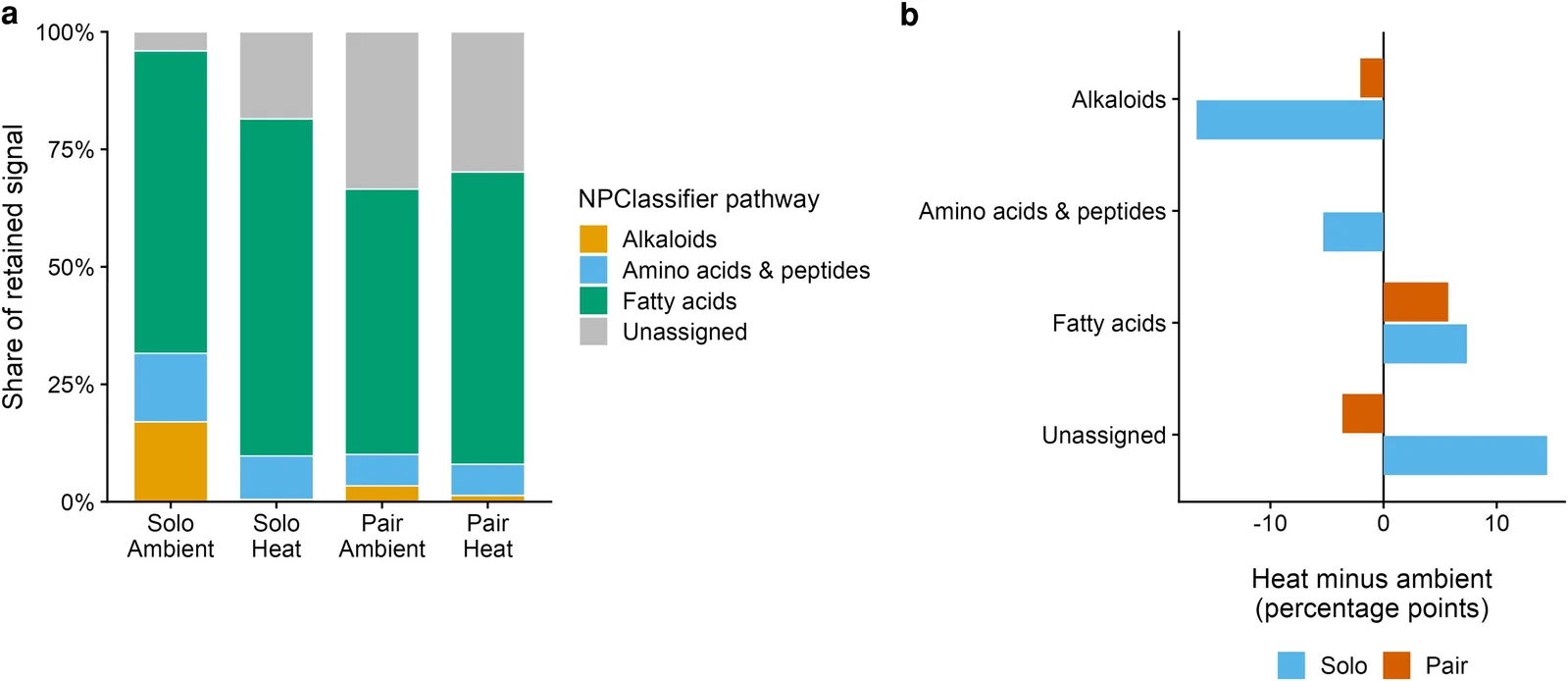
Chemical class composition under social and thermal treatments (Experiment 2). (a) Mean share of retained signal per NPClassifier pathway for each treatment × temperature combination (box means). (b) Change in pathway share from ambient to heat (percentage points) in solo and paired boxes.

Within paired rhizoboxes, composition did not differ between the inner zone between the two plants and the outer zones lateral to the pair (*R*^2^ = 0.04, *F*_1,22_ = 0.85, *P* = .53). Total signal, richness and Shannon diversity did not differ either (paired *t*_5_ ≤ 1.36, all *P* ≥ .23), and no feature differed after FDR correction (Supplementary Figure S2; Supplementary Tables S3–S5). With three paired rhizoboxes per temperature, the minimum differences detectable at 80% power were 13 850 for total signal, 10 features for richness and 1.24 for Shannon diversity.

## Discussion

### A modular platform for rhizosphere metabolomics in soil

This study presents a low-cost, 3D-printed rhizobox system for spatially resolved rhizosphere metabolomics under realistic soil conditions. Across two experiments, the system supported consistent plant growth, reproducible root development along the imaging surface, and reliable recovery of plant-associated metabolites from soil extracts. Soil-containing controls overlapped planted samples in unfiltered data, which is why a feature-wise, blank-informed filter is required.

A key advantage of the system is the integration of non-destructive root imaging with targeted rhizosphere sampling. By visually confirming root presence before soil collection, the approach reduces spatial uncertainty compared to conventional pot experiments. At the same time, it retains soil structure and microbial activity, which are absent from hydroponic or agar-based systems. By combining soil realism with analytical control, the rhizobox provides an experimental bridge between molecular-scale metabolomics and genetic and ecological experimentation on root exudation, plant–plant interactions, and plant–microbe interactions. This framework enables metabolite-level measurements to be directly linked to plant genotypes, phenotypes, and interaction contexts under realistic soil conditions. Comparisons between soil-based rhizosphere metabolomes and hydroponic exudate profiles may further help disentangle compounds that are directly exuded from those shaped by soil-mediated transformation, offering a path towards integrating molecular, genetic, and ecological perspectives in future studies.

### Performance across contrasting plant growth forms

Validation using *A. thaliana* and *P. australis* demonstrated that the rhizobox system accommodates species with markedly different root architectures and growth strategies. Despite differences in root morphology i.e. fine and highly branched roots in *A. thaliana* versus coarser, clonal rhizomes in *P. australis*, both species established readily and produced consistent rhizosphere metabolite profiles across biological replicates. Accordingly, part of the higher total rhizosphere signal in *A. thaliana* likely reflects greater root development, in addition to species-specific differences in metabolite composition. This highlights the flexibility of the system and supports its applicability beyond small model plants.

Species-specific chemical signatures under identical soil and analytical conditions are consistent with known differences between these taxa. *Arabidopsis thaliana* rhizoboxes had higher total signal, richness and diversity and a larger share of fatty acids. This agrees with reports that *A. thaliana* exudation is dominated by low-molecular-weight metabolites involved in nutrient acquisition, signalling and microbial recruitment (Badri and Vivanco 2009, Strehmel et al. 2014). The phenolic and allelopathic compounds reported for *P. australis* (Bais et al. 2003, Rudrappa et al. 2007) were not resolved among its few retained features. Several *P. australis* samples contained no retained feature, indicating that its rhizosphere signal was close to the detection limit of the filter at this early developmental stage.

### Social context as a dominant structuring factor of rhizosphere chemistry

Applying the rhizobox to a factorial manipulation of social context and temperature showed that intraspecific neighbour presence was the main driver of rhizosphere metabolome structure in *A. thaliana*. Social context explained about a quarter of compositional variation among rhizoboxes and was associated with higher feature richness and diversity, whereas short-term heat had no detectable effect.

Notably, total metabolite signal did not differ between solo and paired plants, suggesting that neighbour effects reflect redistribution of chemical investment across metabolites rather than increased overall release. At the level of individual features, relatively few metabolites responded strongly to social context after correction for multiple testing, consistent with the limited size of curated soil metabolite datasets. Because paired rhizoboxes contained two root systems in the same soil volume, part of the higher richness may reflect greater root mass rather than neighbour identity; designs that match root mass, or that use root length as a covariate (Data S7), are needed to separate the two.

These findings align with a growing body of literature showing that plant–plant interactions influence root allocation, architecture, and physiology (Brooker et al. 2008, De Kroon et al. 2009, Novoplansky 2009, Cahill et al. 2010, Subrahmaniam et al. 2018), but extend this work by demonstrating that social context leaves a detectable chemical signature in soil. Importantly, the rhizobox system enables such effects to be quantified while maintaining soil structure and microbial activity, which are essential for realistic chemical diffusion and transformation.

Zone-resolved sampling was possible only in paired rhizoboxes, giving three boxes per temperature, so the inner–outer comparison could detect only large differences. Two non-exclusive explanations fit the absence of a zone effect: diffusion and microbial turnover may homogenize soluble metabolites across the few millimetres between zones, or real gradients may be steeper than the sampling footprint. Finer sampling (a thinner soil layer or a sampling grid) or more rhizoboxes would distinguish these. For studies in which the spatial contrast is itself the hypothesis, we recommend six to eight rhizoboxes per treatment.

### Heat stress responses and their interaction with social context

Short-term heat had no detectable effect on composition, richness or diversity at the rhizobox level, and only one feature, in solo rhizoboxes, responded after FDR correction. Heat shifted pathway shares more in solo than in paired rhizoboxes (Fig. 5b), but these descriptive shifts should be treated as hypotheses. An acute 2-h exposure at 45°C followed by 2 h of recovery may be too short for changes in exudation to accumulate in the soil metabolite pool. The rhizobox allows stress intensity, duration and timing to be varied to test when abiotic stress reshapes chemically mediated plant interactions.

### Methodological considerations and future directions

While the rhizobox system offers substantial advantages, several limitations should be acknowledged. The small soil volume constrains experiment duration and so far limits the amount of material available for parallel analyses, such as microbiome sequencing. Soil heterogeneity and microbial turnover may influence metabolite stability, and the acute heat-stress regime applied here captures short-term responses rather than longer-term acclimation. Future studies could extend this framework by incorporating time-series sampling, isotope labelling to trace metabolite origin and turnover, or combined metabolomic–microbiome approaches. The modular design also allows straightforward adaptation to multi-genotype combinations, sequential planting, or repeated stress exposures, enabling explicit tests of genotype-by-genotype and legacy effects mediated through soil chemistry. The internal dimensions (97 × 94 × 10 mm) suit *A. thaliana* and a desktop printer bed but restrict larger-rooted species. Width and height can be increased (Supplementary Figure S3), but for zone-resolved sampling the soil layer should remain about 10 mm, so that the imaged root plane and the sampled soil stay in register; deeper cavities, such as the 25 mm layer of the enlarged design, suit root imaging and rhizosphere sampling without zone resolution. Beyond about 150 mm of free window span, a central rib or thicker PMMA prevents deflection, and larger soil volumes need longer to equilibrate in water content. For deep-rooted species, height should be increased before width.

## Conclusion

The 3D-printed rhizobox system enables reproducible cultivation, imaging, and spatially resolved rhizosphere sampling under realistic soil conditions. Validation with *A. thaliana* and *P. australis* demonstrated stable growth and robust recovery of species-specific rhizosphere metabolite profiles. Application to *A. thaliana* under contrasting social and thermal contexts showed that intraspecific neighbour presence is a dominant driver of rhizosphere metabolome structure, whereas short-term heat stress had no detectable effect. By making rhizosphere chemistry experimentally tractable without sacrificing soil realism, this protocol bridges molecular metabolomics and ecological experimentation. It provides a framework for mechanistic studies of how plants modify their chemical environment in response to neighbours and stress, advancing our understanding of below-ground interactions in plant communities.

## Supplementary Material

plag055_Supplementary_Data

## Data Availability

The rhizobox print file and dimensioned schematic (Data S1, S2), R analysis scripts (Data S3), extraction protocol (Data S4), MZmine and SIRIUS processing parameters (Data S5), sample metadata (Data S6), root traits (Data S7), and the MZmine feature tables and SIRIUS/CANOPUS annotation outputs needed to rerun the analyses are openly available under a CC BY 4.0 licence at Zenodo: https://doi.org/10.5281/zenodo.18630193. Processed results are also provided as Supporting Information (Supplementary Tables S1–S5).
