# Supplementary material for: A transparent window into the rhizosphere: a simplified workflow for spatially resolved soil metabolomics
Source: AoB Plants. 2026 Oct 30;18(6):plag055. doi: 10.1093/aobpla/plag055 (PMC13725918; doi:10.1093/aobpla/plag055)
Supplement: Supplementary file 1 [file plag055_supplementary_data.zip › Supporting_Information_AoBP-2026-032.pdf]

Supporting Information

A transparent window into the rhizosphere: a simplified workflow for spatially resolved soil metabolomics

Harihar Jaishree Subrahmaniam, Paul Moritz, Phillip J. Becker, Maria Riedner, Simon Thomsen, Ina C. Meier, Kai Jensen

AoB PLANTS, manuscript AOBP-2026-032

Contents of this file

Figure S1. Ordinations of the unfiltered feature tables of both experiments, with and without solvent blanks.
Figure S2. Inner versus outer sampling zones in the paired rhizoboxes of Experiment 2.
Figure S3. An enlarged rhizobox (internal 140 × 180 × 25 mm) filled with a clay soil.
Table S1. Experimental design and replication.
Table S2. Metabolite features retained in Experiment 1, with annotations and species tests.
Table S3. Metabolite features retained in Experiment 2, with annotations, planned contrasts and inner-versus-outer zone tests.
Table S4. Multivariate tests of metabolite composition (PERMANOVA and dispersion).
Table S5. Summary metabolome metrics and univariate tests.
Data S2. Dimensioned rhizobox schematic.
Data S4. Rhizosphere soil extraction protocol.
Data S5. MZmine and SIRIUS processing parameters.

Provided separately

Data S6 (sample-level metadata, CSV), Data S7 (root traits from window images, two CSV files) and an editable workbook of Tables S1–S5 (XLSX) are provided as separate files. Data S1 (rhizobox 3D-print file, STL) and Data S3 (R analysis scripts) are archived at Zenodo (https://doi.org/10.5281/zenodo.18630193), together with the MZmine feature tables and SIRIUS/CANOPUS outputs used as input to the scripts.

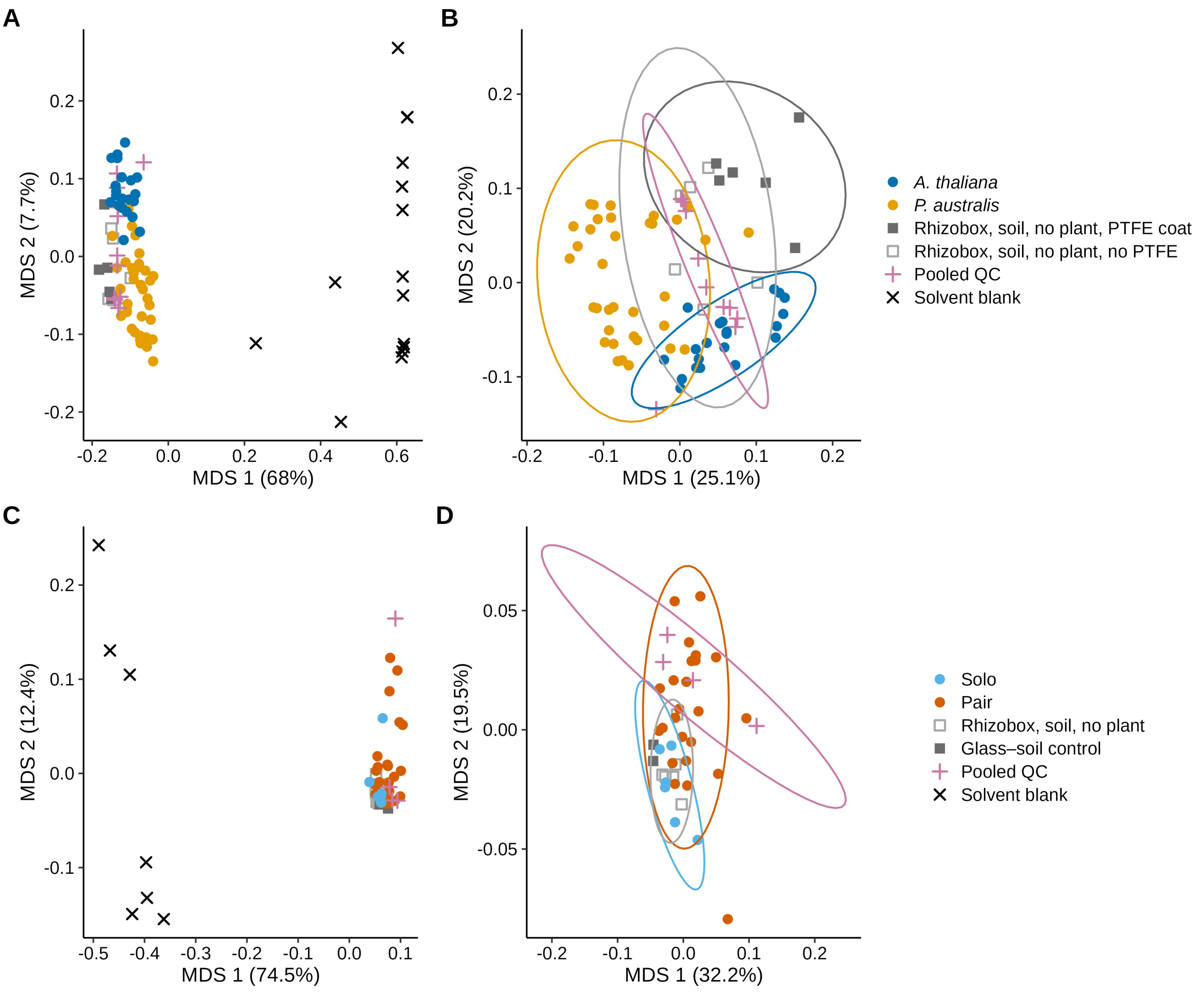

Figure S1. Ordinations of the unfiltered feature tables (Bray–Curtis, metric MDS). (A) Experiment 1, all samples, including solvent blanks. (B) Experiment 1 without solvent blanks, with 95% ellipses per category. (C, D) The same for Experiment 2. Soil-containing controls overlap the planted samples because they share the soil matrix; only solvent blanks separate. This is why a feature-wise filter, rather than a whole-profile criterion, is used.

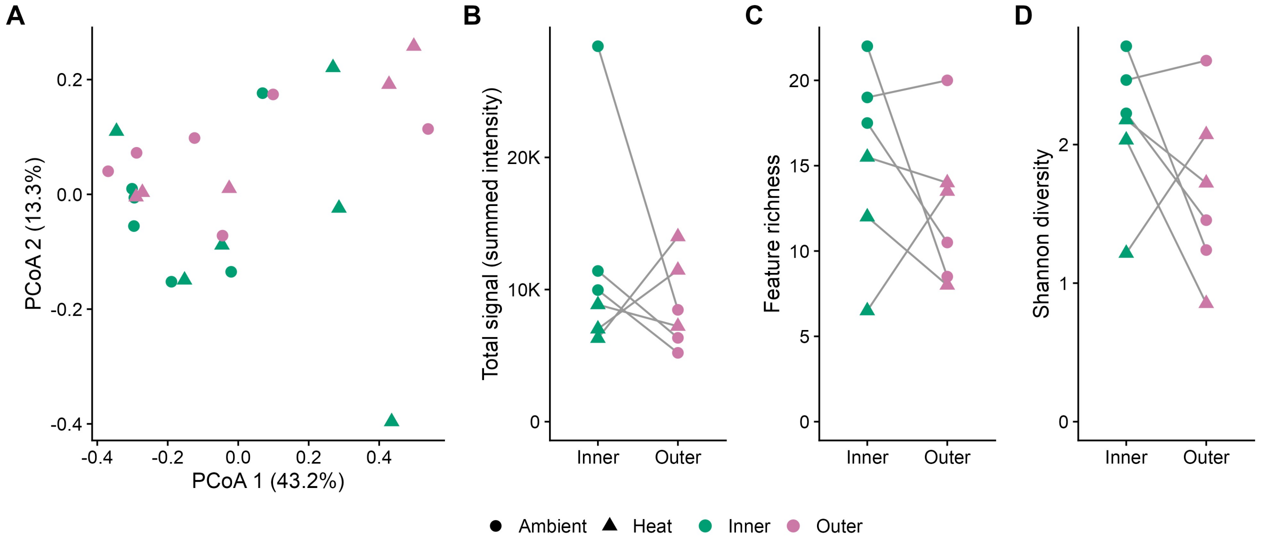

Figure S2. Inner versus outer zones in the paired rhizoboxes of Experiment 2. (A) PCoA (Jaccard) of the 24 zone samples. (B–D) Total signal, richness and Shannon diversity per zone. Lines join the inner and outer means of the same rhizobox. Statistics are in Tables S4 and S5.

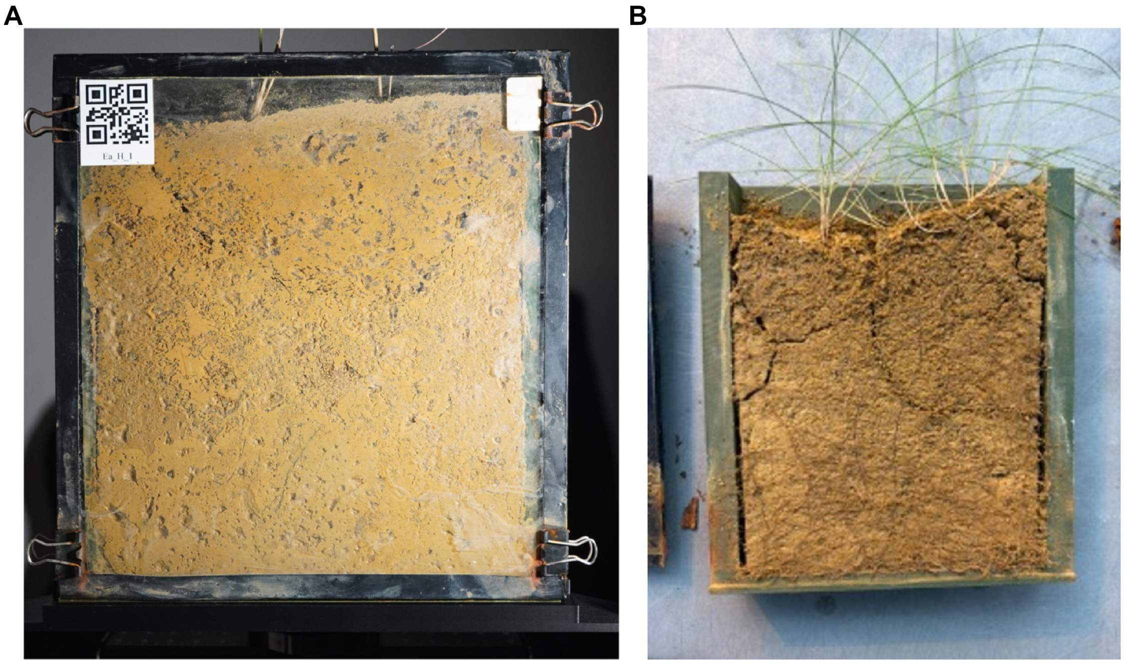

Figure S3. An enlarged rhizobox (internal 140 × 180 × 25 mm; outer 166 × 183 × 31 mm) filled with a clay soil. (A) Front view through the window. (B) Planted with a graminoid.

Table S1. Experimental design and replication. For each experiment, the number of rhizoboxes and samples that entered the analysis, by treatment group, and the number of control samples by control type. The rhizobox is the unit of replication for all between-box tests; samples taken from the same box on different dates, depths or zones are subsamples. Fewer rhizoboxes were analysed than planted owing to plant loss. Sample-level metadata are given in Data S6.

**Experiment**	**Type**	**Group**	**Rhizoboxes analysed**	**Samples analysed**	**Sampling per box**
1	Planted	A. thaliana	4	21	3 dates × 2 depths
1	Planted	P. australis	6	35	3 dates × 2 depths
1	Control	Rhizobox with soil, no plant, with PTFE coat		6	
1	Control	Rhizobox with soil, no plant, no PTFE coat		6	
1	Control	Pooled QC injection		11	
1	Control	Solvent blank		15	
2	Planted	Pair, ambient	3	12	2 inner + 2 outer zones
2	Planted	Pair, heat	3	12	2 inner + 2 outer zones
2	Planted	Solo, ambient	3	3	1 sample
2	Planted	Solo, heat	3	3	1 sample
2	Control	Glass–soil control		2	
2	Control	Pooled QC injection		4	
2	Control	Rhizobox with soil, no plant (ambient)		3	
2	Control	Rhizobox with soil, no plant (heat)		3	
2	Control	Solvent blank		7	

Table S2. Metabolite features retained in Experiment 1 (20 features passing the blank-informed filter: absent from all controls and above the limit of detection in at least three rhizoboxes of one species). Annotations are SIRIUS/CSI:FingerID molecular formulae or names and CANOPUS/NPClassifier pathways and classes; – indicates no confident annotation. Intensities are means of log2(intensity + 1) per rhizobox, averaged over boxes; the species difference was tested with a linear model on rhizobox means (4 A. thaliana and 6 P. australis boxes), with Benjamini–Hochberg false discovery rate (FDR) across features. Features are numbered by ascending m/z.

Feature ID	m/z	RT (min)	Annotation (formula or name)	Adduct	NPClassifier pathway	NPClassifier class	Mean log2 intensity, A. thaliana	Mean log2 intensity, P. australis	Difference (P. australis − A. thaliana)	p	FDR
1	135.0819	14.45	$C_9H_{12}O_2$	$[M - H_2O + H]^+$	Shikimates and Phenylpropanoids	Cinnamic acids and derivatives	4.87	1.74	-3.14	0.022	0.031
2	171.1034	14.56	–	–	Unassigned	–	3.05	0.89	-2.16	0.017	0.026
3	172.1351	13.38	$C_9H_{17}NO_2$	$[M + H]^+$	Amino acids and Peptides	Aminoacids	7.89	2.93	-4.96	0.006	0.014
4	281.1931	17.06	–	–	Unassigned	–	8.10	0.00	-8.10	< 0.001	< 0.001
5	282.2094	17.06	–	–	Unassigned	–	1.39	0.00	-1.39	0.007	0.014
6	299.2038	17.06	$C_{14}H_{28}F_2O_4$	$[M + H]^+$	Terpenoids	Halogenated fatty acids	8.51	0.00	-8.51	< 0.001	< 0.001
7	306.2434	14.55	$C_{19}H_{31}NO_2$	$[M + H]^+$	Alkaloids	Piperidine alkaloids	2.40	0.44	-1.97	< 0.001	0.004
8	367.2131	14.13	$C_{17}H_{31}FO_7$	$[M + H]^+$	Terpenoids	Dicarboxylic acids	0.64	0.65	0.00	0.999	0.999
9	375.3257	14.60	$C_{25}H_{42}O_2$	$[M + H]^+$	Terpenoids	Apocarotenoids (β-)	4.08	1.68	-2.41	0.017	0.026
10	383.2509	1.18	$C_{18}H_{34}BN_3O_3S$	$[M + H]^+$	Amino acids and Peptides	Tripeptides	5.27	0.00	-5.27	< 0.001	< 0.001
11	399.2549	14.01	$C_{22}H_{35}FO_5$	$[M + H]^+$	Terpenoids	Pregnane steroids	0.84	1.16	0.32	0.623	0.656
12	421.2371	14.03	$C_{22}H_{35}FO_5$	$[M + Na]^+$	Terpenoids	Halogenated fatty acids	0.69	1.27	0.58	0.369	0.410
13	504.5191	15.17	–	–	Unassigned	–	0.00	1.03	1.03	0.045	0.056
14	533.3893	14.79	$C_{27}H_{50}F_2N_4O_4$	$[M + H]^+$	Alkaloids	Tripeptides	0.00	0.99	0.99	0.113	0.132
15	605.6041	15.04	$C_{36}H_{78}N_4O$	$[M + Na]^+$	Fatty acids	N-acyl amines	6.38	0.00	-6.38	< 0.001	< 0.001
16	607.5106	14.40	–	–	Unassigned	–	3.39	0.64	-2.75	0.002	0.004
17	617.6042	14.93	$C_{39}H_{76}N_4O$	$[M + H]^+$	Fatty acids	N-acyl amines	4.39	0.00	-4.39	0.011	0.020
18	659.5268	14.56	$C_{36}H_{70}N_2O_8$	$[M + H]^+$	Polyketides	Open-chain polyketides	3.79	1.03	-2.76	0.006	0.014
19	681.5090	14.56	–	–	Unassigned	–	4.44	1.84	-2.60	0.029	0.039
20	751.5043	15.35	$C_{42}H_{70}O_{11}$	$[M + H]^+$	Polyketides	Macrotetrolides	4.67	0.52	-4.14	0.001	0.004

Table S3. Metabolite features retained in Experiment 2 (28 features: absent from every solvent blank, glass–soil control and temperature-matched unplanted-soil control, and above the limit of detection in at least three rhizoboxes under one temperature). Contrast columns give the difference in log2 intensity (second group minus first; for example pair minus solo) with the FDR in brackets. Contrasts are on rhizoboxes (3 vs 3); because paired boxes carry four zone samples and solo boxes one, each contrast was computed on one randomly drawn zone sample per paired box and the median over 999 draws is shown. A blank cell means the feature was not detected in either group of that contrast. Inner vs outer zone: paired test across the six paired boxes (inner minus outer). Annotation columns as in Table S2.

Feature ID	m/z	RT (min)	Annotation (formula or name)	Adduct	NPClassifier pathway	Ambient: pair vs solo	Heat: pair vs solo	Solo: heat vs ambient	Pair: heat vs ambient	Inner vs outer zone
1	150.1127	0.60	C6H15NO3	[M + H]+	Unassigned	6.08 (0.249)	-2.57 (0.449)	10.47 (< 0.001)	2.89 (0.789)	2.33 (0.611)
2	194.1387	0.59	C8H19NO4	[M + H]+	Fatty acids	3.36 (0.641)	-1.56 (0.460)	7.86 (0.588)	2.87 (0.768)	1.17 (0.710)
3	196.1696	12.57	–	–	Unassigned	5.03 (0.228)	2.36 (0.399)		-2.30 (0.769)	2.68 (0.631)
4	210.1853	12.57	–	–	Unassigned	4.98 (0.266)	2.73 (0.399)	-2.29 (0.588)	-4.61 (0.644)	1.65 (0.708)
5	224.2008	12.57	–	–	Unassigned	4.64 (0.355)	2.70 (0.423)		-2.29 (0.754)	0.19 (0.958)
6	226.1800	13.51	C13H23NO2	[M + H]+	Amino acids and Peptides	-3.19 (0.560)	-2.96 (0.566)	-0.06 (0.989)	-0.02 (0.779)	2.35 (0.710)
7	250.1777	13.52	C10H23N3O4	[M + H]+	Amino acids and Peptides	-4.99 (0.489)	-2.82 (0.566)	0.61 (0.989)	0.58 (0.748)	2.23 (0.648)
8	256.3570	13.03	–	–	Unassigned	5.38 (0.228)	2.74 (0.405)		-2.24 (0.751)	1.43 (0.710)
9	256.4294	13.03	–	–	Unassigned	5.70 (0.214)	4.63 (0.305)		-2.34 (0.748)	0.12 (0.963)
10	256.6388	13.03	–	–	Unassigned	5.45 (0.214)	2.80 (0.390)		-2.59 (0.698)	-0.22 (0.958)
11	258.1699	5.28	C10H26N3OP	[M + Na]+	Alkaloids	2.33 (0.552)		-2.28 (0.588)	-4.95 (0.588)	-0.85 (0.710)
12	261.1307	5.45	C7H21N5O2P	[M + Na]+	Alkaloids	-1.25 (0.783)	-2.33 (0.463)	-1.19 (0.989)	-2.27 (0.778)	-0.32 (0.919)
13	273.7229	12.27	–	–	Unassigned	3.25 (0.419)	2.40 (0.423)		-2.71 (0.648)	1.37 (0.710)
14	298.3103	14.14	C19H39NO	[M + H]+	Fatty acids	3.56 (0.427)	2.38 (0.423)		-2.89 (0.647)	0.96 (0.710)
15	306.2766	13.88	C18H37NO	[M + Na]+	Fatty acids	0.47 (0.269)	0.51 (0.266)	-0.19 (0.588)	-0.19 (0.748)	0.20 (0.611)
16	306.2789	12.89	–	–	Unassigned	3.72 (0.427)			-3.72 (0.621)	1.51 (0.611)
17	309.2982	13.38	C11H34N9O	[M + H]+	Alkaloids	5.11 (0.278)	4.96 (0.271)		-0.20 (0.831)	2.15 (0.611)
18	330.2766	13.38	C20H37NO	[M + Na]+	Fatty acids	3.12 (0.505)	3.14 (0.493)	-0.44 (0.989)	-1.28 (0.806)	-0.06 (0.978)
19	399.8409	12.28	–	–	Unassigned	5.65 (0.242)	5.12 (0.313)	-2.31 (0.588)	-3.07 (0.680)	1.52 (0.611)
20	530.4925	13.03	–	–	Unassigned	5.14 (0.262)	2.76 (0.405)		-1.97 (0.805)	2.65 (0.631)
21	538.4788	13.03	–	–	Unassigned	8.12 (0.002)	5.37 (0.271)		-2.96 (0.694)	1.57 (0.611)
22	538.9803	13.03	–	–	Unassigned	4.78 (0.247)	2.82 (0.390)		-0.70 (0.790)	0.88 (0.826)
23	551.5503	13.34	C35H70N2O2	[M + H]+	Fatty acids	7.44 (0.010)	2.90 (0.399)		-4.68 (0.646)	1.51 (0.710)
24	578.4925	12.57	–	–	Unassigned	7.72 (0.002)	5.08 (0.305)		-2.57 (0.700)	0.82 (0.708)
25	591.4399	12.28	–	–	Unassigned	5.38 (0.242)	5.01 (0.305)	-2.29 (0.588)	-2.59 (0.690)	-0.32 (0.919)
26	658.1207	13.03	C25H33NO15S	[M + K]+	Unassigned	3.73 (0.427)	2.33 (0.423)		-1.42 (0.676)	1.03 (0.710)
27	718.1206	12.57	C26H34AsN7O10	[M + K]+	Amino acids and Peptides	3.77 (0.427)	2.43 (0.423)		-1.41 (0.676)	1.02 (0.710)
28	718.6223	12.57	C42H79N5O4	[M + H]+	Amino acids and Peptides	3.75 (0.427)	2.40 (0.423)		-1.43 (0.676)	1.06 (0.710)

Table S4. Multivariate tests of metabolite composition. PERMANOVA (vegan::adonis2, sequential sums of squares, 9,999 permutations) on Jaccard distances of presence–absence profiles unless stated, and tests of multivariate dispersion (betadisper/permutest). Between-box factors (species, social context, temperature) are tested on one profile per rhizobox; within-box factors (date, depth, zone) on samples with permutations restricted within rhizobox. In Experiment 2 the between-box test uses one randomly drawn zone sample per paired box so that paired and solo boxes carry equal sampling effort; the medians of 999 draws are shown. df = numerator, denominator.

Experiment	Test	Term	df	Pseudo-F	R^2	p	Replicate unit	Note
1	PERMANOVA, Jaccard (presence per box)	Species	1, 8	4.82	0.376	0.005	rhizobox (n = 10)	
1	PERMANOVA, Bray-Curtis (box-mean log2 intensity)	Species	1, 8	7.68	0.490	0.005	rhizobox (n = 10)	
1	Dispersion (betadisper), Jaccard per box	Species	1, 8	5.90		0.036	rhizobox (n = 10)	
1	PERMANOVA, Jaccard, permutations within box	Sampling date	2, 30	1.21	0.051	0.238	sample (n = 46) within box	
1	PERMANOVA, Jaccard, permutations within box	Depth (upper vs lower)	1, 30	0.50	0.010	0.859	sample (n = 46) within box	
1	PERMANOVA, Jaccard, permutations within box	Species x date	2, 30	1.10	0.046	0.330	sample (n = 46) within box	
2	PERMANOVA, Jaccard, one sample per box (median of 999 draws)	Social context	1, 8	4.01	0.267	0.011	rhizobox (n = 12)	p < 0.05 in 100% of draws
2	PERMANOVA, Jaccard, one sample per box (median of 999 draws)	Temperature	1, 8	1.78	0.122	0.126	rhizobox (n = 12)	p < 0.05 in 0% of draws
2	PERMANOVA, Jaccard, one sample per box (median of 999 draws)	Social x temperature	1, 8	0.66	0.045	0.657	rhizobox (n = 12)	p < 0.05 in 0% of draws
2	PERMANOVA, Jaccard, permutations within box (paired boxes)	Zone (inner vs outer)	1, 22	0.85	0.037	0.526	sample (n = 24) within box	
2	PERMANOVA on box centroids (paired boxes)	Temperature (paired boxes only)	1, 4	3.94	0.497	0.100	rhizobox (n = 6)	

Table S5. Summary metabolome metrics and univariate tests. Upper part: means ± SE across rhizoboxes of the per-sample total signal (sum of retained-feature intensities), feature richness (number of retained features detected) and Shannon diversity (of relative feature intensities); sample values were first averaged within each box. Lower part: linear models on rhizobox means (Experiment 1: species; Experiment 2: two-way ANOVA of social context × temperature) and paired t-tests of inner versus outer zones in the paired boxes of Experiment 2, with the minimum difference detectable at 80% power (MDD80).

**Experiment**	**Group**	**Rhizoboxes**	**Samples**	**Total signal**	**Feature richness**	**Shannon diversity**
1	A. thaliana	4	21	8,843 ± 1,057	7.8 ± 0.2	1.70 ± 0.05
1	P. australis	6	35	1,918 ± 1,109	1.9 ± 0.5	0.46 ± 0.14
2	Solo, ambient	3	3	4,706 ± 1,670	4.7 ± 1.2	1.02 ± 0.22
2	Solo, heat	3	3	8,098 ± 1,293	5.0 ± 0.6	1.16 ± 0.17
2	Pair, ambient	3	12	11,633 ± 3,423	16.2 ± 1.7	2.12 ± 0.21
2	Pair, heat	3	12	9,144 ± 612	11.6 ± 1.6	1.68 ± 0.15
**Tests**				**Statistic**	**p**	
1	Total: Species			F(1, 8) = 18.30	p = 0.003	
1	Richness: Species			F(1, 8) = 80.73	p < 0.001	
1	Shannon: Species			F(1, 8) = 44.76	p < 0.001	
2	Total: Social context			F(1, 8) = 3.84	p = 0.086	
2	Total: Temperature			F(1, 8) = 0.05	p = 0.830	
2	Total: Social x temperature			F(1, 8) = 2.09	p = 0.186	
2	Richness: Social context			F(1, 8) = 46.78	p < 0.001	
2	Richness: Temperature			F(1, 8) = 2.66	p = 0.141	
2	Richness: Social x temperature			F(1, 8) = 3.54	p = 0.097	
2	Shannon: Social context			F(1, 8) = 17.91	p = 0.003	
2	Shannon: Temperature			F(1, 8) = 0.63	p = 0.450	
2	Shannon: Social x temperature			F(1, 8) = 2.23	p = 0.174	
2	Total signal: inner vs outer (paired)	6		t(5) = 0.81	p = 0.452	MDD80 = 13850
2	Richness: inner vs outer (paired)	6		t(5) = 1.05	p = 0.342	MDD80 = 10.05
2	Shannon: inner vs outer (paired)	6		t(5) = 1.36	p = 0.231	MDD80 = 1.24

Data S2. Dimensioned rhizobox schematic.

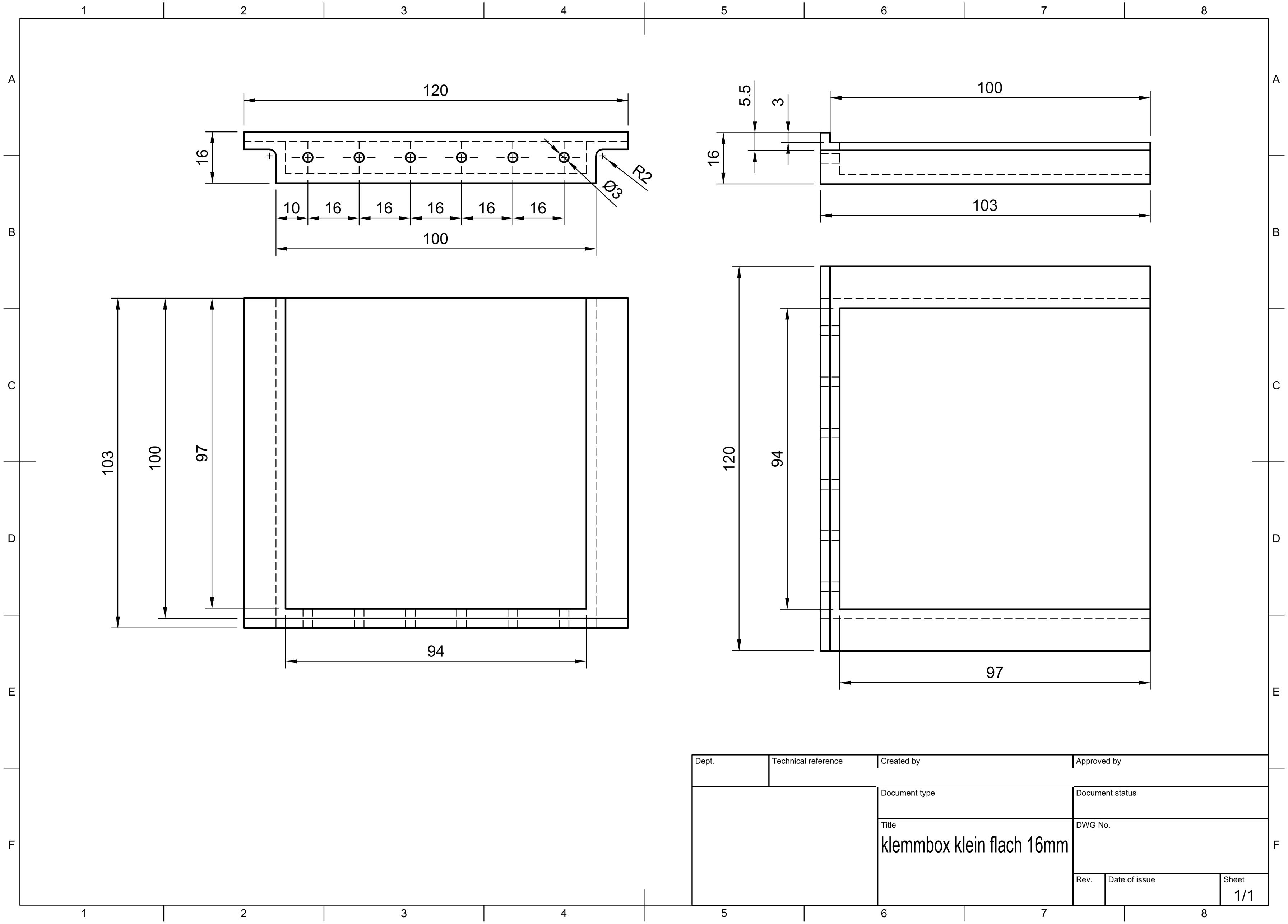

Data S4. Rhizosphere metabolite extraction protocol

Materials Needed

- **Solvent:** LC-MS grade Methanol:Water:Formic Acid (80:19.9:0.1 v/v/v)
- **Soil:** Rhizosphere soil (soil tightly adhering to roots or sampled immediately below root zone)
- **Equipment:** Vortex, centrifuge (≥10,000 g), sonicator (optional), 0.22 µm PTFE filters, -20°C freezer
- **Optional:** SPE (C18 or HLB cartridges) for humic acid removal

Protocol Steps

Step	Action	Notes
1. **Sample Prep**	Collect 1–2 g **freeze-dried rhizosphere soil**	Fresh is OK if frozen immediately; avoid long room temp exposure
2. **Add Solvent**	Add 5–10 mL of cold **MeOH:H_2O:Formic Acid (80:19.9:0.1)**	Use LC-MS grade solvents; pre-chill to 4°C
3. **Vortex & Sonicate**	Vortex 30 sec → Sonicate 10–15 min in cold water bath	Enhances metabolite diffusion
4. **Incubate**	Place on ice or 4°C for 30–60 min (gentle shaking optional)	Allows thorough extraction
5. **Centrifuge**	Spin at 10,000–14,000 × g for 10–15 min at 4°C	Pellet soil debris
6. **Collect Supernatant**	Transfer supernatant to new tube	Handle gently to avoid disturbing pellet
7. **Optional Repeat**	Re-extract pellet with fresh solvent; combine supernatants	Increases recovery, esp. for low-abundance metabolites
8. **Filter**	0.22 µm PTFE filter to remove particulates	Essential for LC-MS
9. **Dry or Concentrate**	Dry under N_2 stream or lyophilize if needed	Helps if sample is too dilute
10. **Reconstitute**	In 50:50 MeOH:H_2O (or LC buffer) for injection	~100–200 µL depending on concentration needs

Data S5. MZmine and SIRIUS parameters

Analysis of mzML files

Raw LC-MS data files (mzML format) were processed using **MZmine** for feature detection, alignment, and MS/MS export, followed by **SIRIUS** for molecular formula prediction, compound class annotation, and structural elucidation. All analyses were performed in positive ionization mode.

MZmine workflow

A) Project setup

MZmine (version 4.7.29) was launched, and a project directory with sufficient disk space was defined using the **"Change"** option in the project settings. All intermediate and output files generated during processing were stored in this directory.

B) Feature detection and alignment using the MZmine Wizard

The **MZmine Wizard** was used for standardized configuration of peak detection, alignment, and MS/MS export.

Data tab

- All mzML files were imported by dragging them into the wizard interface or via the **"Select files"** button.

HPLC tab

- Smoothing: **Enabled**
- Stable ionization across samples: **Enabled**
- Crop retention time: **0.5–60 min**
- Maximum peaks per chromatogram: **15**
- Minimum consecutive scans: **4**
- Approximate feature full width at half maximum (FWHM): **0.10 min**
- Retention time tolerance (intra-sample): **0.08 min**
- Retention time tolerance (sample-to-sample): **0.40 min**

QTOF tab

- Ion mode: **Positive**
- Intensity filter type: **Absolute intensity**
- MS1 noise threshold: **1.0×10^4**
- MS2 noise threshold: **1.0×10^2**
- Minimum feature height: **1.0×10^3**
- m/z tolerance (scan-to-scan): **0.0050 m/z or 20 ppm**
- m/z tolerance (intra-sample): **0.0015 m/z or 3 ppm**
- m/z tolerance (sample-to-sample): **0.0040 m/z or 8 ppm**

Filters tab

- Original feature list: **Removed**
- Minimum samples per aligned feature: **2**
- Require presence of ^{13}C isotope: **Disabled**

Annotation tab

- Default settings were retained.

DDA tab

- Apply spectral networking (FBMN / IIMN): **Enabled**
- Export path: user-defined results directory
- Export for molecular networking: **Enabled**
- Export for SIRIUS: **Enabled**
- Export annotation graphics: **Disabled**

C) Batch execution

- Wizard settings were optionally saved using **“Save presets”**.
- A processing batch was generated by clicking **“Create batch”**, followed by **“OK”** to load the batch queue.
- Feature detection and alignment were executed using **“Run batch”**.
- Upon completion, an MS/MS export file (*_sirius.mgf) was generated in the output directory for downstream SIRIUS analysis.

SIRIUS workflow

A) Data import

SIRIUS (version 6.3.3) was launched, and a new project directory was created using **“New”**. The *_sirius.mgf file generated by MZmine was imported by drag-and-drop. Successful import resulted in visualization of all detected MS/MS spectra within the SIRIUS interface.

B) SIRIUS computation parameters

Molecular formula prediction and annotation were initiated using **“Compute all”** with the following settings:

General settings

- Instrument: **QTOF**
- Mass accuracy: **10 ppm**
- Fix formula for detected lipids: **Enabled**
- Fallback adducts (positive mode):
    - $[M + H]^+$
    - $[M + Na]^+$
    - $[M + K]^+$ (optional)
    - $[M + C_2H_6OS + H]^+$ (optional; used when MeOH or DMSO was present)

Molecular formula generation

- Strategy: **De novo + bottom-up**
- Apply element filter to: **De novo**
- Allowed elements: **C, H, N, O, P**
- Autodetected elements: **B, S, Cl, Se, Br**
- De novo prediction applied below **m/z 400**

Database searches

- Enabled databases: **PubChem, COCONUT, ChEBI**, and others as available
- PubChem fallback search: **Enabled**
- Confidence mode: **Approximate**

ZODIAC

- ZODIAC was enabled to improve molecular formula confidence.
- Default ZODIAC parameters were used.

CANOPUS (compound class prediction)

- CANOPUS classification was enabled using default score thresholds for prediction of compound superclass, class, and subclass.

C) Final computation

After verification of all parameters, the analysis was initiated by clicking **"Compute"**. Molecular formula predictions, compound class annotations (CANOPUS / NPClassifier), and structural candidates were exported and integrated with curated feature tables for downstream statistical analyses.
